# Pomegranate Peel Extract Is a Potential Alternative Therapeutic for Giardiasis

**DOI:** 10.3390/antibiotics10060705

**Published:** 2021-06-11

**Authors:** Asmaa M. El-Kady, Iman A. M. Abdel-Rahman, Samer S. Fouad, Khaled S. Allemailem, Taghrid Istivan, Sheren F. M. Ahmed, Al Shaimaa Hasan, Heba A. Osman, Hatem A. Elshabrawy

**Affiliations:** 1 Department of Medical Parasitology, Faculty of Medicine, South Valley University, Qena 83523, Egypt; asmaa.elkady@med.svu.edu.eg; 2 Department of Pharmacognosy, Faculty of Pharmacy, South Valley University, Qena 83523, Egypt; emanabdelraheem@svu.edu.eg; 3 Veterinary Clinical Pathology, Qena University Hospital, South Valley University, Qena 83523, Egypt; samer.saad80@yahoo.com; 4 Department of Medical Laboratories, College of Applied Medical Sciences, Qassim University, Buraydah 51452, Saudi Arabia; K.allemailem@qu.edu.sa; 5 Biosciences and Food Technology, School of Science, RMIT University, Melbourne, Bundoora, VIC 3083, Australia; Taghrid.istivan@rmit.edu.au; 6 Department of Pathology, Faculty of Medicine, Sohag University, Sohag 82524, Egypt; shery.pathology@yahoo.com; 7 Department of Medical Pharmacology, Faculty of Medicine, South Valley University, Qena 83523, Egypt; drelshimaa.hassan@med.svu.edu.eg; 8 Tropical Medicine, Gastroenterology and Hepatology Department, Faculty of Medicine, South Valley University, Qena 83523, Egypt; hebaahmed198098@yahoo.com; 9 Department of Molecular and Cellular Biology, College of Osteopathic Medicine, Sam Houston State University, Conroe, TX 77304, USA

**Keywords:** pomegranate, *Punica granatum*, *Giardia lamblia*, giardiasis, IL-6, NO, TNF-α, cysts, trophozoites, intestine, villi, caspase-3

## Abstract

Giardiasis is a major diarrheal disease affecting approximately 2.5 million children annually in developing countries. Several studies have reported the resistance of *Giardia lamblia* (*G. lamblia*) to multiple drugs. Therefore, identifying an effective drug for giardiasis is a necessity. This study examined the antiparasitic effect of *Punica granatum* (pomegranate) and evaluated its therapeutic efficacy in rats infected with *G. lamblia*. In vitro study showed high efficacy of pomegranate peel ethanolic extract in killing *G. lamblia* cysts as demonstrated by eosin vital staining. We showed that treating infected rats with pomegranate extract resulted in a marked reduction in the mean number of *G. lamblia* cysts and trophozoites in feces and intestine respectively. Interestingly, the number of *G. lamblia* trophozoites and cysts were significantly lower in the pomegranate extract-treated group compared to the metronidazole-positive control group. Moreover, pomegranate extract treatment significantly induced nitric oxide (NO) and reduced serum IL-6 and TNF-α, compared to infected untreated rats. Histological and scanning electron microscopy (SEM) examination of the jejunum and duodenum of pomegranate extract-treated animals confirmed the antiparasitic effect of the extract, and demonstrated the restoration of villi structure with reduction of villi atrophy, decreased infiltration of lymphocytes, and protection of intestinal cells from apoptotic cell death. In conclusion, our data show that the pomegranate peel extract is effective in controlling *G. lamblia* infections, which suggests that it could be a viable treatment option for giardiasis.

## 1. Introduction

Giardiasis, caused by *Giardia lamblia* (*G. lamblia*), has been considered the second-most common cause of infectious diarrhea in humans after viral diarrhea [1]. According to the World Health Organization (WHO), *G. lamblia* is one of the most common intestinal parasites affecting Eastern Mediterranean and African children and is now included in the WHO’s Neglected Disease Initiative [2]. It causes approximately 2.5 million cases of childhood diarrhea annually in developing countries [3,4]. Prevalence of giardiasis among children in Egypt increased to 27.3% [5,6]. *G. lamblia* is typically transmitted by exposure to contaminated water and food and could remain asymptomatic or become symptomatic with a wide range of clinical symptoms [7,8,9]. In a natural setting, the rate of symptomatic infection varies from 5% to 70%. In 70–75% of symptomatic patients, abdominal cramping, bloating, and flatulence have been reported [5,6]. Other complications of giardiasis include weight loss, malabsorption, and growth retardation [10,11]. In developing countries, giardiasis is a common cause of chronic diarrhea and growth retardation in children [12].

In giardiasis, intestinal colonization by the parasite has been identified to cause microvillus shortening and villous flattening or atrophy [13]. These pathological changes, possibly in combination with other pathological mechanisms, such as reduction in intestinal disaccharidase and protease activity, may be the direct causes of giardia-associated diarrhea. *Giardia*-induced apoptosis of enterocytes, by activation of caspases, is a key mechanism of the giardiasis-induced enteric pathology [14]. *G. lamblia* trophozoites have been shown to induce apoptosis in enterocytes both in vitro and in chronic giardiasis patients [15,16,17]. *Giardia-*induced apoptosis was reported to be mediated via intrinsic and extrinsic apoptosis pathways. The intrinsic pathway starts by activation of pro-apoptotic proteins such as caspase-3 and -9, leading to apoptotic cell death [15,16]. Moreover, it is well documented that *G*. *lamblia* infections trigger the extrinsic apoptotic pathway by activating caspase-8 [16]. In addition, recent studies have established that immune-mediated apoptosis induced by Fas or TNF-α is responsible for increased epithelial permeability in giardiasis [18].

Many drugs, including metronidazole, are currently used in the treatment of human giardiasis. Metronidazole was initially used in treating *Trichomonas vaginalis* infections [19]. Since then, its therapeutic applications have been extended to include treatment of amoebiasis, giardiasis, and anaerobic bacterial infections [20,21,22]. However, a number of side effects have been reported following metronidazole treatment, including headache, vertigo, nausea, and a metallic taste in the mouth [23,24]. In addition, pancreatitis, neurotoxicity, and neutropenia have been attributed to nitroimidazole anti-infectives, including metronidazole [21,23]. Metronidazole has been identified to be mutagenic in bacteria and carcinogenic in mice and rats at high doses over longer periods [25,26]. Clinical studies have reported the resistance of *G. lamblia* to different medications [19,22]. For that reason, studies have focused on testing the efficacy of herbal drugs in treating giardiasis [27].

Pomegranate (*Punica granatum*) is a plant that has been used in folk medicine for the treatment of various human diseases [28]. The constituents of pomegranate include high levels of hydrolyzable tannins (punicalins and punicalagins), ellagic acid (a component of ellagitannins), and gallic acid (a component of gallotannins), with well-known antioxidant and antimicrobial activities [29]. Additionally, pomegranate is widespread in the Mediterranean region and is well known for its anti-inflammatory effect due to its high content of antioxidants [28]. Furthermore, pomegranate is a potential antifungal and it is casually used in the treatment of *Trichophyton rubrum* [30]. Pomegranate exhibits several other pharmacological activities, including anthelmintic effects on various intestinal trematodes, nematodes, and cestodes in addition to its anti-amoebic, antimalarial, and anticoccidial effects [31,32,33,34,35,36,37].

In this study, we aimed to investigate the therapeutic utility of pomegranate in *G. lamblia* infections. For that purpose, pomegranate peel ethanolic extract was tested for its effectiveness in eliminating the parasite and ameliorating the disease in a rat model of giardiasis.

## 2. Materials and Methods

### 2.1. Plant Material

Fresh Egyptian pomegranate fruits were collected from an agricultural field near South Valley University. The taxonomic identification and authentication of the collected plants were conducted by the Department of Pharmacognosy, Faculty of Pharmacy, South Valley University, Egypt. A voucher specimen of the plant (code: Pg.90) was kept in the herbarium, Department of Pharmacognosy, Faculty of Pharmacy, South Valley University, Egypt. The pomegranates were thoroughly washed to get rid of any debris; the peels were removed, cut into small pieces, and dried in an air circulatory tray drier at 60 °C for 6 h. Dried peels were ground using a laboratory disc mill and the powder was passed through a 20 mesh sieve. The powder was stored at room temperature until used for extraction.

### 2.2. Preparation of Pomegranate Extract

Pomegranate ethanolic extract was prepared as previously described by Almoniem et al. [28]. Briefly, dried pomegranate peels were powdered. The ethanolic extract was prepared by maceration of 150 g of peels powder in 750 mL of 70% ethanol for 16 h at room temperature, and then filtered. Filtrate was evaporated to dryness in a rotary evaporator under reduced pressure, to obtain the crude ethanolic extract. The dry crude extract residue (11 g) was stored at 4 °C for subsequent preparation of the required doses.

### 2.3. Collection of Cysts

*G. lamblia* cysts were obtained from stools of patients who complained of diarrhea and presented to outpatient clinics of the University Hospital at South Valley University, Qena, Egypt. Positive samples were processed, washed with saline, centrifuged, and viability of cysts was confirmed using 0.1% eosin vital staining. We then counted the number of cysts in phosphate-buffered saline (PBS). The process was repeated until we finally obtained a concentration of 10,000 cysts/mL of PBS, which is the required dose for the animal infection experiments.

### 2.4. In Vitro Test of the Effect of Pomegranate Ethanolic Extract on G. lamblia Cysts

We added 2 mL of different concentrations (1, 10 and 50 mg/mL) of pomegranate ethanolic extract to 1 mL of *G. lamblia* cysts suspension in sterile test tubes. We used 2 mL of metronidazole at a concentration of 50 μg/mL as the positive control [38], whereas the untreated *G. lamblia* cyst suspension was used as the negative control. All test tubes were thoroughly mixed, incubated at 37 °C for 5, 10, 30 or 60 min. At the end of each time interval, *G. lamblia* cysts viability was assessed using 0.1% eosin vital staining and a hemocytometer at 400× magnification. The percentage of dead cysts/high-power field (HPF) was calculated according to Al-kaissi [39].

### 2.5. Animal Experiments

Animal experiments were performed in the Animal Facility at the Parasitology Department, Faculty of Medicine, South Valley University, Qena, Egypt. Thirty-two 3–4-week-old male golden rats, weighing 150–200 g, were used for the present study. Animals were initially examined daily for the presence of any parasitic infections. To establish the *G. lamblia* infection, 10,000 cysts were administered to animals [40]. In our study, animals were divided equally into four groups with eight rats in each group. The first group represents the uninfected and untreated control group, whereas the second group included animals that were infected with *G. lamblia* but did not receive any treatment (negative control). The third and fourth animal groups consisted of infected animals that were treated for 7 days with either ethanolic extract of pomegranate at a dose of 300 mg/kg/day or metronidazole (positive control) at a dose of 15 mg/kg/day, respectively [32,41]. Treatments started on the third day post-infection and all animals were sacrificed 3 days after the end of treatments [41].

### 2.6. Assessment of the In Vivo Efficacy of the Extract

#### 2.6.1. Determination of Cysts and Trophozoite Counts

To ensure infection of animals, fecal samples were examined microscopically for the presence of cysts on the third day post-infection. Three days after the end of treatments, the number of cysts were counted in stools of all animals/high-power field [42]. Briefly, 1 g of freshly collected feces from each rat was completely homogenized in 10 mL of formal saline, then cysts were counted using hemocytometer at 400× magnification, as previously described [42]. After sacrificing the animals, the mean number of *G. lamblia* trophozoites in the intestine was determined based on five different high-power fields (400×)/animal, as previously described [43].

#### 2.6.2. Quantification of Proinflammatory Cytokines and NO

Sera were prepared from blood samples and stored at −20 °C for analysis. We quantified the levels of IL-6 and TNF-α in animals’ sera using the IL-6 and TNF-α ELISA Kits, according to the manufacturer’s instructions (KOMA BIOTECH INC., Seoul, South Korea, Cat. No. K0331229 and Cat. No. K0331196, respectively) [44]. The NO levels were determined by measuring NO end products (NOx) using Biodiagnostic colorimetric assay kit.

#### 2.6.3. Histological Studies

##### Hematoxylin and Eosin Staining

Formalin-fixed paraffin-embedded intestinal tissue blocks were used to prepare 4-µm-thick sections. These sections were deparaffinized in xylene, rehydrated in decreasing alcohol concentrations (100%, 80%, 70%, and 50%; 1 min each), and rinsed in distilled water followed by running tap water for 3–5 min. The slides were stained with hematoxylin for 5–7 min, washed in running tap water, and then stained with eosin for 3–5 min followed by running tap water. The slides were dehydrated in increasing concentrations of ethanol (50% ethanol, 70%, 80%, and 100%; 1 min each), cleared in xylene, and mounted with dibutyl phthalate polystyrene xylene (DPX) and cover-slipped. Slides were then examined using light microscopy, at 400× magnification, to evaluate intestinal villi atrophy and lymphocyte infiltration in different animal groups. We recorded the number of animals/group with either absent, mild, moderate, or marked villi atrophy. Similarly, we counted the number of animals/group with mild, moderate, or severe intestinal tissue lymphocyte infiltration.

##### Immunohistochemistry

Four-µm-thick sections were cut and mounted on saline-coated glass slides, which were subsequently incubated overnight at room temperature to optimize adhesion of the tissues onto the slides. The slides were deparaffinized in xylene for 20 min, rehydrated in decreasing concentrations of alcohol (100% for 5 min, then 2 min in each of 80%, 70%, and 50%), and rinsed in distilled water. Endogenous peroxidase activity was blocked by incubating the tissue sections in 0.6% hydrogen peroxide (H_2_O_2_) for 10 min. The tissue sections were then washed twice with PBS, and antigens were retrieved by boiling the sections twice in Tris/EDTA buffer (pH = 9.0) in a microwave oven at mid–high power for 10 min. Next, sections were left to cool down to room temperature for 30 min. After washing twice with PBS, tissue sections were treated with superblock. Tissue sections were then rinsed twice with PBS and incubated with rabbit recombinant monoclonal Caspase-3p12 antibody (clone No. EPR16888, Abcam, Cambridge, MA, USA) at a concentration of 1:2000 overnight at room temperature. Excess reagent was discarded, tissue sections were rinsed twice in PBS containing 0.05% Tween-20 (PBS-T), then incubated with HRP-conjugated goat anti-rabbit secondary antibody (Vivantis Technologies, Selangor Darul Ehsan, Malaysia) at a dilution of 1:5000 for 1 h at 4 °C. After washing twice with PBS-T, the color was developed by incubating sections with 0.05% diaminobenzidine (DAB) and 0.01% H_2_O_2_ for 3 min. Finally, the sections were counterstained with hematoxylin, washed in running water, dehydrated in graded ascending series of alcohols (70%, 80%, 90%, and 100%), cleared in xylene for 5 min, and mounted with DPX, cover-slipped, and imaged at 400× magnification.

##### Immunohistochemical Scoring

The estimated average staining intensity of caspase-3 in intestinal tissues was scored as follows: 0 (no signal), 1 (weak signal), 2 (moderate signal) and 3 (strong signal). The percentage of stained cells at each intensity level was graded as 0 (<5%), 1 (5–25%), 2 (26–50%), 3 (51–75%), or 4 (>75%). The intensity score and percentage of positive cells were then added to yield the final scores (0–7). A score of 0 indicated absence of caspase-3, scores ≤4 were considered low expression, whereas scores > 4 reflected high expression [45].

#### 2.6.4. Scanning and Transmission Electron Microscopy (SEM)

Tissues from the duodenum and proximal jejunum of the sacrificed rats were immediately fixed in 3% cold glutaraldehyde in phosphate-buffered saline (PBS, pH 7.4) for 2 h. The tissues were then washed 3× with PBS for 10 min each. Next, they were fixed in 2% osmium tetroxide in water for 2 h, rinsed 3× in water for 5 min each, then dehydrated in ascending ethanol concentrations (once for 10 min in 30%, 50%, 70%, 80%, 90% ethanol, and twice in 100% ethanol). The tissues were then dried using critical-point drying, with liquid CO_2_, at 34 °C and pressure of 1200 psi. Finally, tissues were mounted on stainless steel holders, sputter-coated with a thin layer of gold, and imaged by scanning electron microscope (JEOL JSM-5500 LV, JEOL, Tokyo, Japan). SEM examination was carried out in the electron microscopy unit, South Valley University, Qena, Egypt, according to Hayat1981 [46].

### 2.7. Statistical Analysis

The collected data were analyzed using the Statistical Package for the Social Sciences (SPSS) version 20 for Windows. All values were expressed as means and error bars represent standard deviations. Differences between groups were determined using analysis of variance tests (ANOVA). Chi-square test was used to determine significance when comparing number of animals in different groups with villi atrophy, lymphocyte infiltration, and caspase-3. In all experiments, probability (*p*) values < 0.05 were considered statistically significant.

## 3. Results

### 3.1. Pomegranate Extract Effectively Killed G. lamblia Cysts

**Figure 1:**
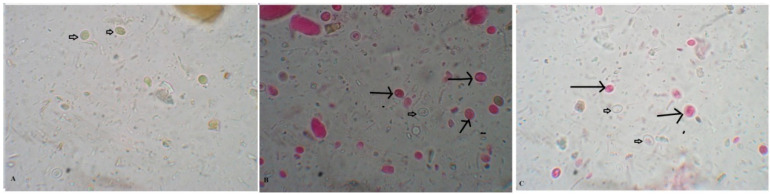
**Pomegranate extract killed *G. lamblia* cysts.** (**A**) Untreated viable *G. lamblia* unstained cysts after staining with 0.1% eosin vital stain (Negative control). Dead cysts (long arrows) and living cysts (short arrows) are shown after incubation with pomegranate extract (50 mg/mL) (**B**) and metronidazole (50 μg/mL) (**C**) for 30 min (400× magnification).

We show that incubation of *Giardia* cysts with different concentrations of pomegranate extract resulted in a significant increase in the percentage of dead *Giardia* cysts, which was dose- and time-dependent (*p* < 0.001). The highest percentage of dead cysts (98%) was seen after 60 min incubation with 50 mg/mL of pomegranate extract (Table 1 and Figure 1).

### 3.2. Pomegranate Extract Reduced Cysts and Trophozoite Counts in G. lamblia-Infected Animals

**Figure 2:**
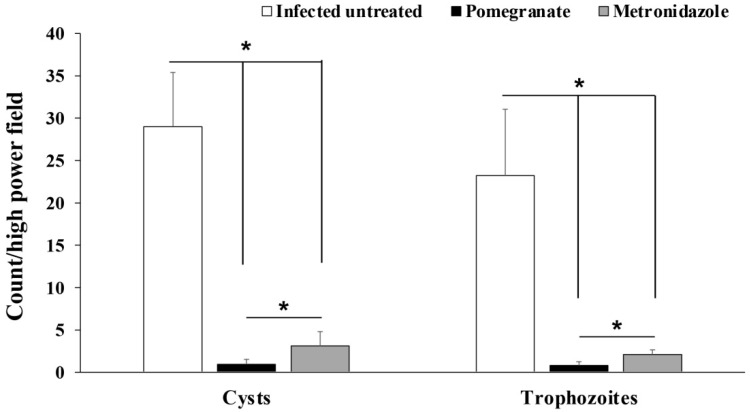
**Effect of pomegranate extract or metronidazole treatments on the number of *G. lamblia* cysts and trophozoites in stool and intestine respectively.** Data are expressed as means with error bars representing SD (n = 8) and were analyzed using ANOVA. Asterisks (*) indicate a significant difference in the numbers of cysts or trophozoites in treated groups compared to the infected untreated group (*p* = 0.003 and 0.001 for cysts and trophozoites, respectively), and a significant difference between the metronidazole- and pomegranate-treated groups; *p* = 0.001.

Examination of fecal samples from pomegranate extract- or metronidazole-treated animals revealed a statistically significant reduction in the number of *G. lamblia* cysts compared to infected untreated rats (*p* = 0.003; Figure 2). Similarly, we found a significant reduction in the number of *G. lamblia* trophozoites in the intestines of pomegranate extract and metronidazole-treated rats compared to the infected untreated rats (*p* = 0.001; Figure 2). Interestingly, the number of cysts and trophozoites in pomegranate extract-treated animals were significantly lower than those in the metronidazole-treated group (*p* = 0.001).

### 3.3. Treatment with Pomegranate Extract Reduced the Levels of Proinflammatory Cytokines and Increased NO Production in G. lamblia-Infected Animals

**Figure 3:**
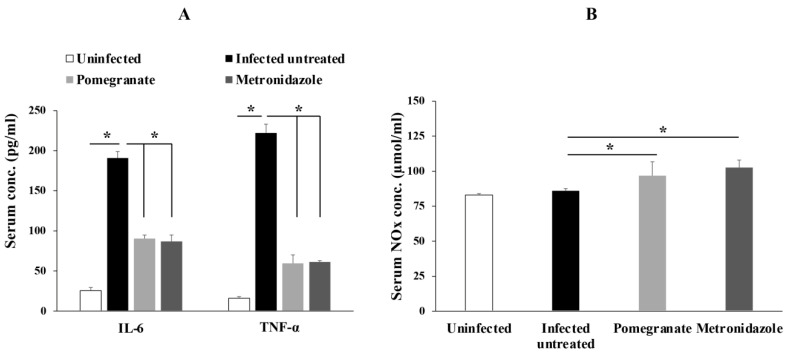
**Pomegranate extract reduced levels of IL-6, TNF-α and increased NO production in animals infected with *G. lamblia*.** Serum levels of IL-6 and TNF- α (**A**) or NOx (**B**) were quantified in different animal groups. Data are expressed as means with error bars representing SD (n = 8), and were analyzed using ANOVA. Asterisks (*) indicate a significant difference (*p* < 0.005).

Next, we aimed to investigate the effect of pomegranate extract on inflammation by measuring the serum levels of proinflammatory cytokines such as IL-6 and TNF-α. Our results showed that animals infected with *G. lamblia* had significantly higher serum levels of IL-6 and TNF-α, which were significantly reduced by pomegranate and metronidazole treatments (Figure 3A; *p* < 0.005).

We also detected higher levels of serum NO end products (NOx), indicative of higher NO production, in pomegranate extract- and metronidazole-treated rats compared to infected untreated rats (Figure 3B; *p* < 0.005).

### 3.4. Treatment with Pomegranate Extract Reduced Lymphocyte Infiltration and Protected the Intestinal Villi from Infection-Induced Pathology

**Figure 4:**
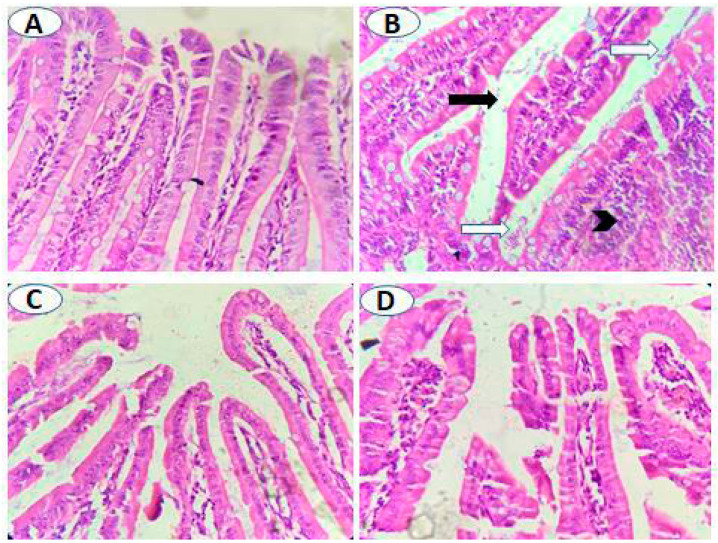
**Pomegranate extract treatment protected intestinal villi from *G. lamblia*-induced atrophy.** (**A**) Normally appearing villi in uninfected animals. (**B**) Villi of infected untreated animals showing atrophy (black arrow), marked infiltration of inflammatory cells mainly lymphocytes in lamina propria (black arrowhead) and numerous flagellated *G. lamblia* (white arrows). Nearly normally appearing intestinal villi with reduced lymphocyte infiltration and absence of *G. lamblia* trophozoites in both metronidazole- (**C**) and pomegranate-treated animals (**D**). Magnification is 400×.

Histological examination of the small intestine of the uninfected untreated animals showed normal intestinal histology, whereas variable degrees of villi atrophy (mostly moderate-to-marked atrophy) were seen in significant numbers of infected untreated animals (Figure 4A,B, and Table 2; *p* = 0.001). Treatment with metronidazole resulted in a significant reduction in villi atrophy (absent to mild) (Figure 4C and Table 2; *p* = 0.001). Similarly, pomegranate treatment significantly restored villi structure with 50% of animals with no atrophy, 25% with mild atrophy, and 25% with moderate atrophy (Figure 4D and Table 2; *p* = 0.001). Additionally, variable degrees of lymphocyte infiltration were observed in the lamina propria of infected untreated animals with the majority (62.5%) showing marked lymphocyte infiltration compared to uninfected animals (Figure 4A,B, and Table 3; *p* = 0.005). However, both metronidazole and pomegranate treatments significantly reduced the lymphocyte infiltration into lamina propria (75% of animals showed mild lymphocyte infiltration) (Figure 4C,D, and Table 3; *p* = 0.005). All the previous results imply that pomegranate treatment is effective in restoring villi and reducing inflammation caused by *G. lamblia* infection.

### 3.5. Pomegranate Extract Treatment Protected Intestinal Tissue from Apoptotic Cell Death Following G. lamblia Infection

**Figure 5:**
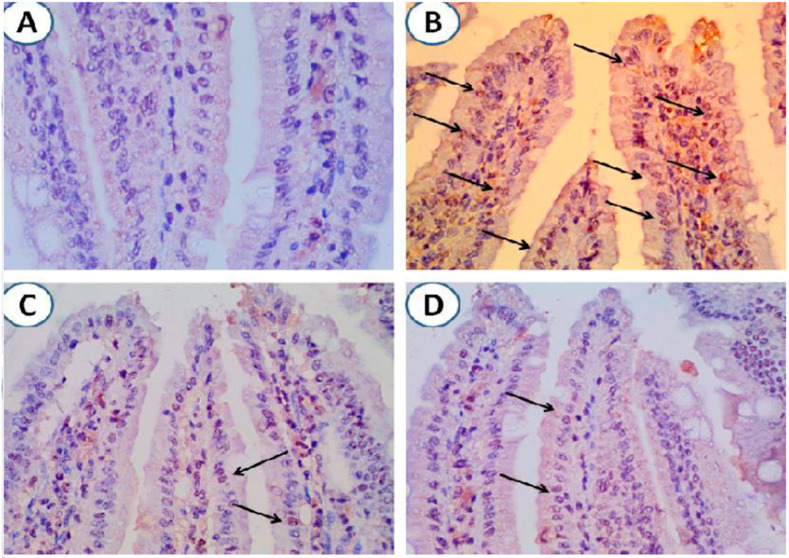
**Treatment with pomegranate-protected intestinal cells from infection-induced apoptosis.** (**A**) Intestinal tissue from uninfected animals with no expression of caspase-3. (**B**) Intestinal tissue from *G. lamblia*-infected untreated animals showing marked expression of caspase-3 (arrows). (**C**) and (**D**). Minimal expression of caspase-3 in intestinal tissues of infected animals treated with pomegranate extract or metronidazole respectively (arrows). Magnification is 400×.

Immunohistochemical staining of caspase-3 in intestinal tissues of animals from different groups showed absence of caspase-3 in 50% of uninfected animals, whereas intestines of a significantly higher number of infected untreated animals (62.5%) demonstrated high levels of capsase-3 (Figure 5A,B, and Table 4). Interestingly, pomegranate- and metronidazole-treated animals showed variable levels of caspase-3 with a significantly higher number of animals having low levels (62.5%) (Figure 5C,D, and Table 4; *p* = 0.001). These results indicate that pomegranate extract treatment is effective in preventing apoptotic cell death of intestinal cells that is induced by *G. lamblia* infection.

### 3.6. Scanning Electron Microscopy (SEM) Showed Marked Ability of Pomegranate Extract to Kill G. lamblia Trophozoites and Protect Intestinal Tissue from G. lamblia Infection

**Figure 6:**
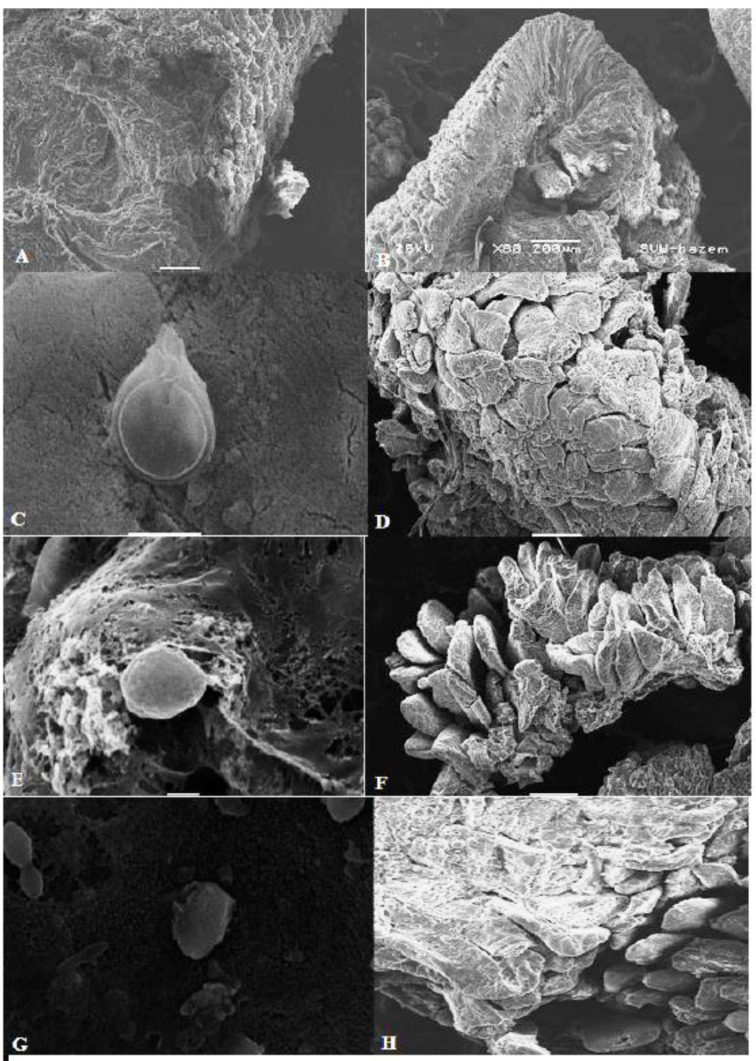
**Scanning electron microscopy (SEM) of the intestine of animals from different groups.** (**A**,**B**) Normal intestinal mucosa of uninfected rats. (**C**) SEM of intestine of infected untreated animal showing normal morphology of *G. lamblia* trophozoite with normal disc and flagella and attachment to the mucosa. (**D**) SEM image showing damage and erosion of the intestinal mucosa in infected untreated animal. (**E**) SEM image of intestinal tissue of metronidazole-treated animal showing distorted morphology of *G. lamblia* trophozoite (loss of flagella and distortion of sucking disc). (**F**) Recovery of the intestinal mucosa after metronidazole treatment. (**G**) SEM image of intestinal tissue of pomegranate-treated animal showing distorted morphology of *G. lamblia* trophozoite (loss of flagella and distortion of sucking disc). (**H**) SEM image showing marked recovery of the intestinal mucosa after pomegranate extract treatment.

Our SEM of the proximal part of the small intestine showed high efficacy of pomegranate extract in damaging *G. lamblia* trophozoites and restoring the structure of intestinal villi. Figure 6A,B shows normal mucosa from uninfected rats with no trophozoites. Examination of intestine from infected untreated animals showed damaged eroded mucosa and harbored *G. lamblia* trophozoites with normal morphology (normal ventral disc, flagella, and attachment to the damaged and eroded intestinal mucosa) (Figure 6C,D). However, SEM images of small intestine from animals that were treated with metronidazole (Figure 6E,F) or pomegranate peel extract (Figure 6G,H) showed recovered intestinal mucosa, and *G. lamblia* trophozoites with distorted morphology (loss of flagella, distortion of sucking disc, and loss of attachment to the intestinal mucosa). These results further confirm the efficacy of pomegranate extract in killing *G. lamblia* trophozoites and protecting intestinal tissue of infected rats.

## 4. Discussion

Giardiasis has become a major global health problem due to resistance of the parasite to currently available drugs. In the current study, we sought to determine the efficacy of pomegranate ethanolic extract in *G. lamblia* infections. Recently, the use of plants and plant-derived products in treatment of different diseases has gained much attention due to their antimicrobial activities and fewer side effects. Also, these herbal products are particularly useful in cases of resistance to conventional medications. Among these plants, pomegranate has been used in folk medicine and is known to possess antiparasitic, antibacterial, antifungal, and antiviral activities [31,32,33,34,35,36,37]. It is well documented that the pomegranate peels contain various active ingredients including tannins, flavonoids, alkaloids and organic acids. Gallagyldilacton, gallic acid, and granatin B were shown to have anti-inflammatory activities [47,48,49]. In addition, studies have demonstrated that Tannins, such as punicalin, punicalagin, pedunculagin, gallic acid and casuarinin, are potent antioxidants [47,49]. Moreover, flavonoids in pomegranate peels have antibacterial, antiviral, antioxidant, anti-inflammatory and anti-neoplastic activities [50,51].

In our current study, we assessed the therapeutic utility of pomegranate as an antigiardiasis agent. We showed that the pomegranate extract is effective in killing *G. lamblia* cysts, which is in agreement with previous studies [31,32,33,34,35,36]. Examination of intestinal mucosa and feces revealed that treatment with pomegranate peel extract markedly reduced the number of trophozoites and cysts in intestinal mucosal and feces of infected animals, respectively, which is consistent with the results of earlier studies [31,32,33,34,35,36]. Moreover, pomegranate peel extract more effectively reduced trophozoites and cysts than the positive control; metronidazole.

Elimination of trophozoites and fecal cysts in response to pomegranate peel extract treatment, in the present study, may be attributed to the production of NO by activated macrophages, which is cytotoxic through a number of different mechanisms [35,52,53]. This mechanism is supported by the higher levels of NO in infected animals that are treated with pomegranate peel extract compared to untreated ones. The lower levels of NO in infected untreated animals have been previously observed by others and could be explained by the ability of the parasite to compete with the host for arginine (substrate for NO) or increase host production of arginase, which reduces arginine availability [54,55]. These mechanisms of limiting NO production are important for trophozoite survival within the intestine [56]. Interestingly, we found that metronidazole-treated rats have slightly higher levels of NO than pomegranate extract-treated animals, which makes pomegranate extract more suitable in the treatment of giardiasis since NO is cytotoxic, not only for the invading parasites, but also for the host cells [57,58].

Our measurement of IL-6 and TNF-α serum levels showed higher levels in infected untreated animals compared to uninfected ones, which is in line with studies which demonstrated major roles of these cytokines in controlling giardiasis [59]. However, treatment with pomegranate peel extract or metronidazole significantly lowered the cytokine levels, as shown in other studies [60,61]. Li et al. reported that higher levels of IL-6 triggered the production of TNF-α during *G. lamblia* infections in mice, and accordingly, TNF-α mRNA levels were lower in IL-6-deficient mice following *Giardia* infection than in wild-type mice [52]. IL-6 has been shown to stimulate B-cell differentiation, antibody production, and T-cell proliferation and modulate IgA response. Therefore, it is not surprising that IL-6-deficient mice exhibit a significant defect in the early control of *G. lamblia* infection [59,62]. Studies have also shown that mice treated with neutralizing anti-TNF-α antibodies or that are genetically deficient in TNF-α failed to control *G. lamblia* infection [44]. We show that rats treated with pomegranate peel extract still have higher levels of IL-6 than uninfected rats, which implies that this extract could be beneficial given that IL-6 plays an important role in intestinal epithelial cell repair [63]. Moreover, TNF-α levels were significantly lower in the pomegranate extract-treated group than in infected untreated group, which is also advantageous since high levels of TNF-α may result in intestinal cell damage [64].

Our histopathological analysis showed that intestines of infected untreated animals exhibit higher numbers of *G. lamblia* trophozoites, marked villi atrophy, and significant lymphocyte infiltration, all of which are consistent with many other studies [41,65]. Damage to the intestinal mucosa in giardiasis involves alterations in the microvilli and epithelial barrier dysfunction [13,66]. In the present study, high levels of TNF-α in infected untreated rats may explain the damage and inflammation of the intestinal villi together with the induction of apoptosis by *G. lamblia* trophozoites [67,68]. We observed significant reduction in the number of trophozoites in the groups treated with pomegranate peel extract or metronidazole, which correlates with marked recovery of the mucosal villi, reduced lymphocyte infiltration, decreased intestinal apoptotic cell death, and pronounced alleviation of the pathological changes to the villous architecture. The previous findings are in line with findings of other studies [41,42,65].

Using SEM, we clearly showed that *G. lamblia* trophozoites in the intestines of the infected untreated animals possess all the main features of normal trophozoites, such as the sucking disk and flagella, and they caused erosion and damage of small intestine [41,42]. Interestingly, treatment with pomegranate peel extract resulted in drastic structural changes in trophozoite structure, including damage of the sucking disk, loss of flagella, and loss of attachment to the intestinal mucosa [41,42]. These observations can be explained by the ability of the extract to damage parasite cell membrane, induce cytoplasm leakage, and parasite swelling, which, in turn, could lead to death of the parasite [42]. SEM examination of the duodenum and proximal jejunum of infected untreated rats showed features of brush border injury [42,69,70]. However, treatment with pomegranate peel extract resulted in marked recovery of the intestinal mucosa. All of these results indicate that pomegranate extract is effective in eradicating *G. lamblia* infection and protecting the intestine from *G. lamblia*-induced pathological damage. We believe that pomegranate extract could be a potential therapeutic option for giardiasis.

## 5. Conclusions

The development of novel drugs to control giardiasis is required because resistance to approved drugs is commonly reported. In this study, we showed that pomegranate peel ethanolic extract has marked efficacy in the treatment of giardiasis in an experimental animal model. Our findings indicate that pomegranate peel extract is effective in killing *G. lamblia* cysts in vitro and significantly reducing the number of cysts and trophozoites in feces and intestine, respectively. Moreover, our data showed that there was marked reduction in levels of proinflammatory cytokines that is accompanied by recovery of the intestinal epithelium and decrease in lymphocyte infiltration into the intestinal tissue. The direct cytotoxic effect of pomegranate extract and the increase in NO production in pomegranate extract-treated animals could be potential mechanisms for the therapeutic effect seen in our study. Our findings suggest that pomegranate is an effective therapeutic for giardiasis and should be considered in cases caused by *G. lamblia*-resistant strains.

## Figures and Tables

**Table 1:** **The effect of increasing concentrations of pomegranate extract on *G. lamblia* cysts’ viability at different exposure times compared to metronidazole (50 μg/mL).** Each treatment was performed in triplicate and asterisks (*) indicate a significant increase in percentage of dead cysts compared to untreated control (*p* < 0.001).

Exposure Time	Pomegranate Extract			Metronidazole	Control
	1 mg/mL	10 mg/mL	50 mg/mL	50 μg/mL	
5 min	51 ± 2.7 *	53 ± 2.1 *	59 ± 1.6 *	59 ± 0.7 *	2 ± 0.2
10 min	63 ± 1.9 *	64 ± 2.1 *	71 ± 1.3 *	60 ± 1.3 *	2 ± 0.4
30 min	71 ± 2.4 *	75 ± 2.6 *	77 ± 1.7 *	77 ± 1.5 *	4 ± 1.4
60 min	84 ± 3.4 *	88 ± 3.1 *	98 ± 1.2 *	91 ± 2.3 *	6 ± 1.8

**Table 2:** **Therapeutic effect of pomegranate extract on intestinal villi atrophy caused by *G. lamblia* infection.** Numbers of rats with absent villi atrophy, mild, moderate, or marked atrophy are presented in addition to their percentages of the total number of rats in each group (8 rats/group). Pomegranate, similar to metronidazole (positive control), significantly reduced villi atrophy compared to infected untreated animals; *p* = 0.001.

Animal Group	Atrophy of Intestinal Villi				*p*-Value
	Absent	Mild	Moderate	Marked	
**Uninfected untreated**	8 (100%)	0 (0%)	0 (0%)	0 (0%)	0.001
**Infected untreated**	0 (0%)	1 (12.5%)	3 (37.5%)	4 (50%)	
**Pomegranate treatment**	4 (50%)	2 (25%)	2 (25%)	0 (0%)	
**Metronidazole treatment**	5 (62.5%)	3 (37.5%)	0 (0%)	0 (0%)	

**Table 3:** **Treatment with pomegranate extract reduced lymphocyte infiltration into intestinal tissue of *G. lamblia*-infected rats.** Number of animals with mild, moderate, or marked lymphocyte infiltration are shown including their percentages of total number of animals/group (n = 8). Pomegranate, similar to metronidazole (positive control), significantly reduced lymphocyte infiltration compared to infected untreated animals; *p* = 0.005.

Animal Group	Lymphocytic Infiltration			*p*-Value
	Mild	Moderate	Marked	
**Uninfected untreated**	6 (75%)	2 (25%)	0 (0%)	0.005
**Infected untreated**	0 (0%)	3 (37.5%)	5 (62.5%)	
**Pomegranate treatment**	6 (75%)	1 (12.5%)	1 (12.5%)	
**Metronidazole treatment**	6 (75%)	2 (25%)	0 (0%)	

**Table 4:** **Treatmentwith pomegranate extract reduced caspase-3 in intestinal tissue of *G. lamblia*-infected rats.** Number of animals with negative, low, or high levels of caspase-3 are shown including their percentages of total number of animals/group (n = 8). Pomegranate, similar to metronidazole (positive control), significantly increased the number of animals with low levels of caspase-3, and reduced the number of animals with high levels of caspase-3 compared to infected untreated animals; *p* = 0.001.

**Animal Group**	**Caspase-3 Levels**			***p*-Value**
	**Negative**	**Low**	**High**	
**Uninfected untreated**	4 (50%)	3 (37.5%)	1 (12.5%)	0.001
**Infected untreated**	1 (12.5%)	2 (25%)	5 (62.5%)	
**Pomegranate treatment**	2 (25%)	5 (62.5%)	1 (12.5%)	
**Metronidazole treatment**	1 (12.5%)	5 (62.5%)	2 (25%)	

## Data Availability

Not applicable.
